# Clinical implementation of gene panel testing for lysosomal storage diseases

**DOI:** 10.1002/mgg3.527

**Published:** 2018-12-11

**Authors:** Alexander Gheldof, Sara Seneca, Katrien Stouffs, Willy Lissens, Anna Jansen, Hilde Laeremans, Patrick Verloo, An‐Sofie Schoonjans, Marije Meuwissen, Diana Barca, Geert Martens, Linda De Meirleir

**Affiliations:** ^1^ Center for Medical Genetics UZ Brussel Brussels Belgium; ^2^ Neurogenetics Research Group, Reproduction Genetics and Regenerative Medicine Research Group Vrije Universiteit Brussel Brussels Belgium; ^3^ Paediatric Neurology Unit, Department of Paediatrics UZ Brussel Brussels Belgium; ^4^ Centre de dépistage néonatal de l’ULB Brussels Belgium; ^5^ Department of Pediatrics Ghent University and Ghent University Hospital Ghent Belgium; ^6^ Department of Pediatric Neurology University Hospital Antwerp (UZA) Antwerp Belgium; ^7^ Department of Medical Genetics University Hospital Antwerp (UZA) Antwerp Belgium; ^8^ Clinic of Pediatric Neurology "Prof. Dr. Alexandru Obregia" Clinical Psychiatric Hospital Bucharest Romania; ^9^ "Carol Davila" University of Medicine and Pharmacy Bucharest Romania; ^10^ VUB Metabolomics Platform, Vrije Universiteit Brussel and Laboratory for Molecular Diagnostics AZ Delta Roeselare Roeselare Belgium

**Keywords:** 4MU-based enzymatic testing, diagnostic testing, gene panel sequencing, lysosomal storage disease, next‐generation sequencing

## Abstract

**Background:**

The diagnostic workup in patients with a clinical suspicion of lysosomal storage diseases (LSD) is often difficult due to the variability in the clinical phenotype. The gold standard for diagnosis of LSDs consists of enzymatic testing. However, due to the sequential nature of this methodology and inconsistent genotype–phenotype correlations of certain LSDs, finding a diagnosis can be challenging.

**Method:**

We developed and clinically implemented a gene panel covering 50 genes known to cause LSDs when mutated. Over a period of 18 months, we analyzed 150 patients who were referred for LSD testing and compared these results with the data of patients who were previously enrolled in a scheme of classical biochemical testing.

**Results:**

Our panel was able to determine the molecular cause of the disease in 22 cases (15%), representing an increase in diagnostic yield compared to biochemical tests developed for 21 LSDs (4.6%). We were furthermore able to redirect the diagnosis of a mucolipidosis patient who was initially suspected to be affected with galactosialidosis. Several patients were identified as being affected with neuronal ceroid lipofuscinosis, which cannot readily be detected by enzyme testing. Finally, several carriers of pathogenic mutations in LSD genes related to the disease phenotype were identified as well, thus potentially increasing the diagnostic yield of the panel as heterozygous deletions cannot be detected.

**Conclusion:**

We show that the implementation of a gene panel for LSD diagnostics results in an increased yield in comparison to classical biochemical testing. As the panel is able to cover a wider range of diseases, we propose to implement this methodology as a first‐tier test in cases of an aspecific LSD presentation, while enzymatic testing remains the first choice in patients with a more distinctive clinical presentation. Positive panel results should however still be enzymatically confirmed whenever possible.

## 1 INTRODUCTION

Lysosomal storage diseases (LSD) affect approximately 1 in 5,000–8,000 worldwide. Currently, mutations in over 50 genes have been reported to disrupt the lysosomal metabolism, leading to a wide spectrum of disease phenotypes including neuropathological effects, musculoskeletal abnormalities, dysmorphia, hepatosplenomegaly, and the occurrence of seizures. For any specific LSD, these multiorgan phenotypes can be present in a varying degree and show significant overlaps across different LSDs. And although most LSDs manifest themselves during early childhood, certain diseases have a genetically specific late‐onset form (e.g., Pompe) or display only more severe effects later in life (e.g., Fabry). Given these challenges, the path to a diagnosis for an LSD in an affected patient can be long and is often unsuccessful. Current diagnostic workflows are predominantly sequential in nature, implying only one test (e.g., urine or biochemical analysis) is initiated depending on the suspicion of the disease.

The use of next‐generation sequencing (NGS) in the clinic during recent years has resulted in a significant increase in diagnostic yield both through a targeted approach with gene panels or untargeted strategies based on whole‐exome sequencing. Here, we propose the incorporation of gene panel testing in the LSD diagnostic workflow. For this purpose, we developed a panel comprising 51 genes which are interrogated based on probe capturing. We investigated 150 patients with a clinical suspicion of an LSD and evaluated this approach compared to classical sequential biochemical testing based on fluorimetric methodologies.

## 2 MATERIALS AND METHODS

### 2.1 Patient selection

The inclusion procedure of the patients in our study was approved by the ethical commission of UZBrussel. During a follow‐up study of 18 months, samples of patients with a suspicion of a lysosomal storage disease were collected and analyzed in our standard diagnostic workflow. In total, the cohort consisted of 150 samples. All pathogenic or potentially pathogenic mutations discovered by the gene panel analysis were confirmed by means of classical Sanger sequencing. Whenever possible, segregation analysis was performed on the patients parents using Sanger sequencing.

### 2.2 Gene panel analysis

Genomic DNA was isolated from blood specimens using the Chemagen DNA kit (PerkinElmer, Shelton, CT) and quantified on a NanoDrop spectrophotometer (ThermoFisher Scientific, Charlotte, NC). Subsequently, the DNA was fragmented with a Covaris ultrasonicator instrument (Woburn, MA). Gene coding regions, as well as the flanking intronic sequences, were captured using SeqCap target enrichment probes (Roche, Basel, Switzerland) according to the manufacturer's protocol. The libraries were paired‐end sequenced (2 × 125 bp) on a HiSeq 1500 machine (Illumina, San Diego, Ca). A minimum coverage of 30× was calculated. FastQ files were analyzed with the SeqNext software package (JSI Medical, Ettenheim, Germany).

### 2.3 Analysis of genomic deletions

Verification of the common 65 kb deletion in the *CTNS* gene was performed according to the methods described by Forestier et al. (1999) and Anikster et al. (1999). Detection of the *CLN3* deletion was carried out based on the methodology of Taschner, Vos, and Breuning (1997).

### 2.4 Biochemical confirmation

The biochemical confirmation was performed both in external accredited laboratories or in‐house. In‐house confirmation was done for IDUA (Anson, Bielicki, & Hopwood, 1992; Clements, Muller, & Hopwood, 1985), GAA (Beratis, LaBadie, & Hirschhorn, 1978) and GBA (Beutler & Kuhl, 1970). Briefly, for IDUA and GBA activity measurements, peripheral blood leukocytes were used and were extracted by adding 2 ml of a 2% dextran solution to 5 ml of blood sample. After 30 min, the supernatant was collected and centrifuged for 6 min at 750 *g*. Subsequently, the cell pellet was washed three times with a 3.6% NaCl solution. Lysis of the cells occurred by resuspending the pellet in cold 3.6% NaCl solution and freeze/thawing the suspension at −80°C. For GAA activity measurement, the same procedure was followed, but skin fibroblasts were used as starting material. Protein content was determined with a standard Lowry assay. For the IDUA activity measurement, the 4MU‐α‐L‐iduronide cyclohexylammonium substrate was used. For GAA activity, the 4MU‐α‐D‐glucopyranoside substrate was used. Measurements were performed under pH4 and pH6 conditions. For GBA, the 4MU‐β‐D‐glucopyranoside substrate was used.

## 3 RESULTS

### 3.1 Gene selection and panel coverage

The composition of our gene panel is shown in Table 1. All 51 tested genes were reported as direct cause of an LSD when mutated in both alleles. For all genes, all exons are covered, so no specific potential hotspots are missed. We did not include genes where a direct connection between mutation and lysosomal storage disease was not thoroughly established. The panel is therefore well suited for diagnostic testing in patients with high a priori probability of LSD based on the clinical phenotype and is not designed as an untargeted screening‐oriented assay.

**Table 1:** Overview of the genes which are investigated with the LSD gene panel

Name disease	Enzyme/protein	Gene	Omim	RefSeq
alpha‐fucosidase	alpha‐L‐fucosidase	*FUCA1*	230,000	NM_000147.4
alpha‐mannosidase	alpha‐D‐mannosidase	*MAN2B1*	248,500	NM_000528.3
Aspartylglucosaminuria	aspartylglucosaminidase	*AGA*	208,400	NM_001171988.1
beta‐mannosidase	beta‐D‐mannosidase	*MANBA*	248,510	NM_005908.3
chitotriosidase	chitotriosidase	*CHIT1*	600,031	NM_003465.2
CLN1	palmitoyl protein thioesterase I	*PPT1*	256,730	NM_000310.3
CLN10	cathepsin D	*CTSD*	610,127	NM_001909.4
CLN2	tripeptidyl peptidase I	*TPP1*	204,500	NM_000391.3
CLN3	ceroid‐lipofuscinosis, neuronal 3	*CLN3*	204,200	NM_001042432.1
CLN5	ceroid‐lipofuscinosis, neuronal 5	*CLN5*	256,731	NM_006493.3
CLN6	ceroid‐lipofuscinosis, neuronal 6	*CLN6*	601,780	NM_017882.2
CLN7	Major facilitator superfamily domain containing 8	*MFSD8*	610,951	NM_152778.2
CLN8	ceroid‐lipofuscinosis, neuronal 8	*CLN8*	600,143	NM_018941.3
Cystinosis	cystinosin (cystine transporter)	*CTNS*	606,272	NM_004937.2
Danon disease	Lysosome‐associated membrane protein 2	*LAMP2*	300,257	NM_001122606.1
Fabry disease	alpha‐galactosidase	*GLA*	300,644	NM_000169.2
Farber lipogranulomatosis	acid ceramidase	*ASAH1*	228,000	NM_177924.4
Galactosialidosis	cathepsin A	*CTSA*	256,540	NM_000308.3
Gaucher disease	beta‐glucosidase	*GBA*	230,800	NM_001005742.2
GM1‐gangliosidosis	beta‐galactosidase	*GLB1*	230,500	NM_000404.3
GM2‐gangliodidosis AB	GM2 activator	*GM2A*	613,109	NM_001167607.1
GM2‐gangliosidosis/Sandhoff	N‐acetyl‐beta‐hexosaminidase A+B	*HEXB*	268,800	NM_000521.3
GM2‐gangliosidosis/Tay‐Sachs	N‐acetyl‐beta‐hexosaminidase A	*HEXA*	272,800	NM_000520.5
Krabbe disease	galactocerebrosidase	*GALC*	245,200	NM_000153.3
Metachromatic leukodystrophy	arylsulfatase A	*ARSA*	250,100	NM_000487.5
MPS1/Hurler syndrome	alpha‐L‐iduronidase	*IDUA*	252,800	NM_000203.5
MPS2/Hunter syndrome	iduronate 2‐sulfatase	*IDS*	309,900	NM_001166550.3
MPS3A/Sanfilippo syndrome A	N‐sulfoglucosamine sulfohydrolase	*SGSH*	252,900	NM_000199.4
MPS3B/Sanfilippo syndrome B	N‐acetylglucosaminidase	*NAGLU*	252,920	NM_000263.3
MPS3C/Sanfilippo syndrome C	heparan‐alpha‐glucosaminide N‐acetyltransferase	*HGSNAT*	252,930	NM_152419.2
MPS3D/Sanfilippo syndrome D	glucosamine (N‐acetyl)‐6‐sulfatase	*GNS*	252,940	NM_002076.3
MPS4A/Morquio syndrome A	galactosamine (N‐acetyl)‐6sulfate sulfatase	*GALNS*	253,000	NM_000512.4
MPS4B/Morquio syndrome B	Beta‐galactosidase‐1	*GLB1*	253,010	NM_000404.3
MPS6/Maroteaux–Lamy syndrome	arylsulfatase B	*ARSB*	253,200	NM_000046.4
MPS7/Sly syndrome	beta‐glucuronidase	*GUSB*	253,220	NM_001293105.1
MPS9	Hyaluronidase‐1	*HYAL1*	607,071	NM_153281.1
Mucolipidose 1	neuraminidase	*NEU1*	256,550	NM_000434.3
Mucolipidosis II alpha/beta or III	N‐acethylglucosamine‐1‐phosphotransferase, alpha/beta subunits	*GNPTAB*	252,500/255,600	NM_024312.4
Mucolipidosis III gamma	N‐acethylglucosamine‐1‐phosphotransferase, gamma subunit	*GNPTG*	255,605	NM_032520.4
Multiple sulfatase deficiency	sulfatase modifying factor 1	*SUMF1*	272,200	NM_182760.3
Niemann–Pick A&B	sphinogmyelinase	*SMPD1*	257,200	NM_001007593.2
Niemann–Pick C1	NPC1	*NPC1*	257,220	NM_000271.4
Niemann–Pick C2	NPC2	*NPC2*	601,015	NM_001363688.1
Papillon–Lefevre syndrome	cathepsin C	*CTSC*	602,365	NM_001814.5
Pompe disease	alpha‐glucosidase	*GAA*	232,300	NM_001079804.2
Prosaposin deficiency	prosaposin	*PSAP*	176,801	NM_002778.3
Pycnodysostosis	cathepsin K	*CTSK*	265,800	NM_000396.3
Salla disease, sialuria	solute carrier family 17 (sodium phosphate cotransporter)	*SLC17A5*	604,369	NM_012434.4
Schindler disease	Nac‐alpha‐D‐galactosaminidase	*NAGA*	609,241	NM_000262.2
Steroid sulfatase	arylsulfatase C	*STS*	308,100	NM_000351.5
Wolman disease, cholesteryl ester SD	acid lipase, cholesterol esterase	*LIPA*	278,000	NM_001127605.2

**Figure 1:**
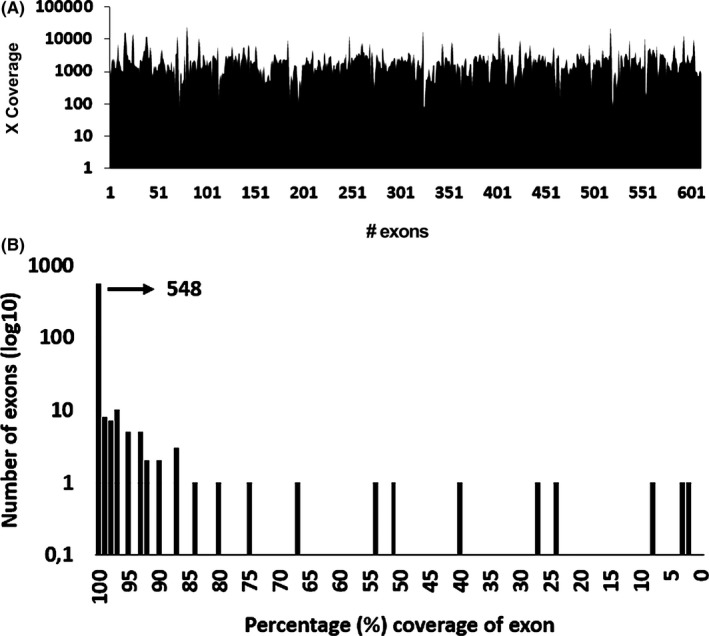
(a) Average coverage of the 609 exons in the LSD gene panel. For each exon, the average coverage was calculated by adding the read depth of each base divided by the total exon length. (b) Graphical overview of percentage of coverage per exon. For each exon, this was calculated by dividing the number of bases with a coverage above 30× by the total number of bases. Five hundred and forty‐eight of a total of 609 exons have a coverage of 100% (fully covered). For three exons, <10% of their nucleotides are covered above 30×

We first assessed its overall analytical performance in terms of depth of coverage in test samples (*n* = 5) that were previously Sanger‐sequenced for LSD‐causing genes. All exons showed an average coverage above 30× (Figure 1a). We also assessed the evenness of coverage within the individual exons (Figure 1b): 79% (548 of 609) of exons were fully covered, with each individual base covered at least 30×. In only 2% (12 of 609) of exons coverage was suboptimal, with 15% or more bases not reaching 30× coverage. On the basis of this data, we conclude that our panel performs sufficiently for the implementation in the clinic.

### 3.2 Sample statistics and diagnostic rate

**Figure 2:**
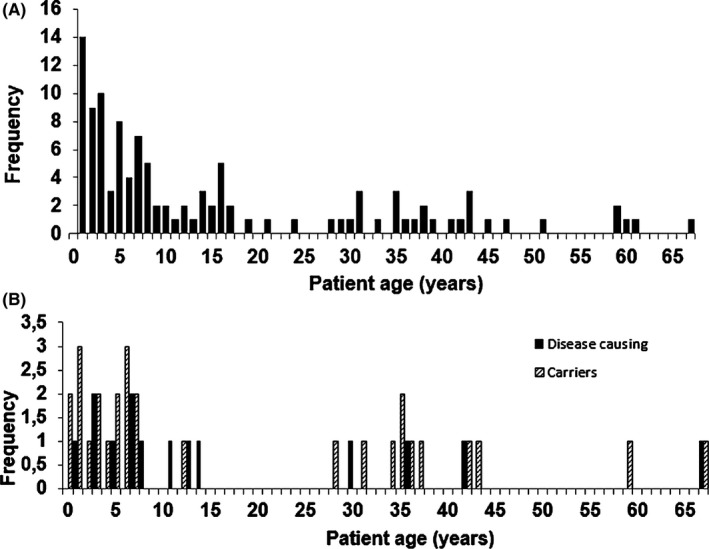
(a) Age distribution of the patients who were tested with the LSD gene panel. (b) Age distribution of the patients in whom a diagnosis was found and where heterozygous mutations were found

Over a period of 18 months, we analyzed 150 samples. As most LSDs present themselves during childhood or adolescence, most patients we analyzed were in this age group. A second important number of patients are in the age group of 30–45 years (Figure 2a). Since children and adolescents are expected to present with a more severe phenotype than the late‐onset patients, we expected the diagnostic success rate to be higher in the younger patient population. Comparing the diagnostic success rate in both patient groups, however, revealed no large differences (Figure 2b).

An overview of the different mutations resulting in a confirmed or likely diagnosis is given in Table 2. In total, we established a diagnosis in 22 of 150 cases, implying a diagnostic yield of 15%. In comparison, we obtained a yield of 4.58% with our previously 4MU‐based biochemical testing panel for 21 LSDs when looking over a period of 30 months (Table 2). Interestingly, four of 22 diagnosed patients were carrier of disease alleles with a pathogenic deletion. For instance, in the *CTNS* and the *CLN3* gene, these deletions are common alleles. In the case of *CTNS*, the 57 kb deletion, comprising exon 1–10, is present in 76% percent of cystinosis patients (Forestier et al., 1999). Likewise, for *CLN3, *the 1.02 kb deletion, spanning exon 7 and 8, is present in 73% of all alleles causing ceroid neuronal lipofuscinosis type 3 (Taschner et al., 1997). These findings stress the need for detection of these deletions into the standard diagnostic LSD pipeline. These deletions cannot be readily detected with our NGS methodology and are detected through standard PCR amplification followed by determination of the amplicon size. Other LSDs where we perform additional deletion analysis are Krabbe and Pompe's disease.

**Table 2:** Overview of the diagnostic rate of the enzymatic 4MU‐based testing over a period of 30 months. A diagnostic yield of 4.58% was obtained

Disease	Enzyme	Detected
Alpha mannosidosis	Alpha‐mannosidase B	1
Fabry	Alpha‐galactosidase	5
Fucosidosis	Fucosidase	1
Gaucher	Acid beta‐glucosidase	4
Hunter	Iduronate‐2‐sulphatase	4
Hurler	Alpha iduronidase	2
Krabbe	Galactocerebrosidase	1
Marotaux‐Lamy	Aryl sulphatase B	4
Metachromatic leukodystrophy	Aryl sulphatase A	6
Morquio A	Galactosamine‐6‐sulphatase	3
Niemann–Pick A/B	Sphingomyelinase	1
Pompe	Acid alpha‐glucosidase	7
Sanfilippo A	Alpha‐N‐sulfoglucosamine sulfohydrolase	2
Sanfilippo B	N‐acetyl‐D‐glucosaminidase	3
Sanfilippo C	Acetyl‐CoA:Alpha‐glucosaminide N‐acetyltransferase	2
Sialidosis I/II	Neuraminidase 1	1
Tay‐Sachs	Hexosaminidase A	2
	Sum	49
	Total of performed analyses	1,069
	Percentage	4.58%
Not performed/detected		
Sanfilippo D	N‐acetylglucosamine‐6‐sulfatase	
Sly disease	Beta‐glucuronidase	
GM1 gangliosidosis	Beta‐galactosidase	
Schindler disease	alpha‐NAc‐galactosaminidase	

In addition, several patients were also found to be carriers of (likely) pathogenic mutations or a variant of uncertain clinical significance (VUS) in one of the LSD genes tested (Table 3). This potentially implies that a large deletion could be responsible for the dysfunctionality of the other allele. However, in a diagnostic setting, investigating the potential role for deletions is only to be considered in case the clinical phenotype of the patient corresponds to the disease spectrum of the gene where a heterozygous mutation is detected. Furthermore, even if a second hit is not found, carrier status implies that the patient and family members can be counseled accordingly. In our study cohort, two patients were detected to be heterozygous for mutations in the *GBA* gene (Table 4). Based on the clinical phenotype, a suspicion for Gaucher's disease could be excluded. However, the connection between heterozygous *GBA* mutations and the development of Parkinson Disease (PD) is starting to be uncovered (Li et al., 2014; Schapira, 2015). Therefore, detection of these mutations has important consequences toward a treatment or follow‐up before the clinical onset of PD of the patient and family members. In line with these findings, we have detected several patients as carrier of a pathogenic mutation in other LSD related genes as well (Tables 3 and 4). For example, the heterozygous c.220C>T mutation was detected in the *SGSH* gene causing mucopolysaccharidosis type 3A (Sanfilippo A). Although only one mutation was detected, the sample was biochemically tested and was found to be SGSH enzyme deficient. Thus, it is possible that the diagnostic yield estimate is higher than the 15% we report here.

**Table 3:** Overview of the variants in patients in whom only one mutation in a specific gene could be detected

Gene	Mutations	Protein	Effect	Literature
*IDUA*	c.1205G>A	p.Trp402*	Pathogenic	Scott, Litjens, Hopwood, and Morris (1992)
*GNPTG*	c.52+2T>G	p.?	Splice effect (5/5 prediction tools)	NA
*NPC2*	c.441+1G>A	p.?	Pathogenic	Staining
*PPT1*	c.363‐4G>A	p.?	Splice effect (0/5 prediction tools)	Kousi, Lehesjoki, and Mole (2012)
*TPP1*	c.509‐1G>C	p.?	Splice effect (5/5 prediction tools)	Dy, Sims, and Friedman (2015)
*GBA*	c.1223C>T	p.Thr408Met	Association with Parkinson disease	Han et al. (2016)
*GBA*	c.222_224delTAC	p.Thr75del	Pathogenic	Koprivica et al. (2000)
*HEXA*	c.1274_1277dupTATC	p.Tyr427Ilefs*5	Pathogenic	Myerowitz and Costigan (1988)
*MAN2B1*	c.418C>T	p.Arg140*	Pathogenic	Riise Stensland et al. (2012)
*CLN8*	c.374A>G	p.Asn125Ser	Pathogenic	Kousi et al., (2012)
*MANBA*	c.1922G>A	p.Arg641His	Pathogenic	Labauge et al. (2009)
*NEU1*	c.1004C>A	p.Pro335Gln	Pathogenic	Bonten (2000)
*ARSB*	c.1334C>T	p.Pro445Leu	Pathogenic	Kantaputra et al. (2014)
*SUMF1*	c.58C>T	p.Leu20Phe	Pathogenic	Cosma et al. (2004)
*NPC1*	c.3614C>A	p.Thr1205Lys	Pathogenic	Park et al. (2003)
*SMPD1*	c.1430C>T	p.Pro477Leu	Pathogenic	Simonaro, Desnick, McGovern, Wasserstein, and Schuchman (2002)
*SMPD1*	c.1460C>T	p.Ala487Val	Pathogenic	Simonaro et al. (2002)
*GNPTAB*	c.1931_1932delinsTG	p.Thr644Met	Pathogenic	Velho et al., (2015)
*SGSH*	c.1159G>A	p.Val387Met	VUS	NA
*CTSC*	c.1319G>A	p.Arg440Gln	VUS	NA
*GNPTAB*	c.1818G>A	p.Met606Ile	VUS	NA
*CLN6*	c.923G>C	p.Ser308Thr	VUS	NA
*ARSB*	c.264G>T	p.Gln88His	VUS	NA
*CTNS*	c.319A>C	p.Asn107His	VUS (+ no deletion detected)	NA
*IDUA*	c.1345C>A	p.His449Asn	VUS	NA
*NEU1*	c.676G>A	p.Asp226Asn	VUS	NA
*AGA*	c.436T>G	p.Leu146Val	VUS	NA

Note

NA: not available; ?: unknown.

**Table 4:** Overview of the mutations detected in the 150 patients in whom a lysosomal storage disease was suspected

Gene	Mutations	Inheritance	Biochemically confirmed
CLN3	c.1222delT;c.1222delT	AR	
CLN3	1,02 kb del;1,02 kb del	AR	
CLN3	1,02 kb del;c.424delG	AR	
CLN6	c.461_463delTCA;c.461_463delTCA	AR	
CTNS	Del 57 kb;Del 57 kb	AR	
GAA	c.2331+2T>A;delE9	AR	Yes
GBA	c.1448T>C;c.1448T>C	AR	Yes
GLB1	c.367G>A;c.817_818delinsCT	AR	
GLB1	c.380G>T;c.1369C>T	AR	Yes
GNPTAB	c.1196C>T;c.3503_3504delTC	AR	Yes
GNPTG	c.377G>A;c.316G>A	AR	
HEXB	c.1082+5G>A;c.1082+5G>A	AR	Yes
IDS	c.998C>T (male patient)	XR	
IDUA	c.1598C>G;c.1598C>G	AR	
IDUA	c.46_57del;c.46_57del	AR	Yes
LIPA	c.894G>A;c.1024G>A	AR	Yes
MAN2B1	c.2248C>T; c.2248C>T	AR	Yes
MFSD8	c.881C>A;c.881C>A	AR	
MFSD8	c.77delT;c.77delT	AR	
NPC1	c.306T>G;c.1691C>A	AR	Yes
NPC2	c.441+1G>A;c.441+1G>A	AR	Yes
SGSH	c.220C>T;?	AR	Yes

Notes

All positive cases found in the panel analysis have been clinically confirmed and all samples which were subjected to biochemical analysis were found to be in the pathological range.

AR: autosomal recessive; XR: X‐linked recessive; ?: unknown.

Below, we describe two cases with an unspecific phenotype where implementation of the gene panel resulted in a diagnosis:

1. A patient with an initial suspicion of galactosialidosis (based on clinical signs and enzyme testing with borderline decreased beta‐galactosidase and absent neuraminidase activity) was found to have two pathogenic mutations in the *GNPTAB* gene, namely c.1196C>T and c.3503_3504delTC, causing mucolipidosis II/III, while no mutations in *CTSA* were present. *GNPTAB *codes for the alpha and beta subunit of the GlcNAc‐1‐phosphotransferase enzyme which catalyzes the first step of the mannose‐6‐phosphate (M6P) tagging of lysosomal enzymes, allowing these to bind to the M6P receptor present on the trans‐Golgi network (Ghosh, Dahms, & Kornfeld, 2003; Qian et al., 2015). This interaction leads to the correct targeting of the enzymes to the lysosomes. As a result, patients affected with mucolipidosis II/III display a reduced activity of multiple enzymes. For instance, mucolipidosis type III is often referred to as pseudo‐Hurler polydystrophy (Coutinho, Prata, & Alves, 2012).
2. A consanguineous couple presented at consultation with their two children, a 7‐year‐old boy and an 8‐year‐old girl, both displaying a neurodegenerative disease course after having obtained normal developmental motoric and verbal milestones. At the age of 5 years, cognitive stagnation was followed by regression in both. The girl developed refractory epileptic seizures at the age of six and myoclonic periods of absence at the age of seven. The boy started to display periods of absence at the age of 6.5 years. The two sibs were furthermore affected by cerebellar atrophy, retinal abnormalities on electroretinograms, and showed signs of dysmetria. Initial genetic analysis for mitochondria‐related diseases did not show any pathogenic alterations. However, LSD panel analysis revealed the two children were homozygous for the c.77delT, p.Leu26Ter mutation in the *MFSD8* gene (alternatively *CLN7*), causing neuronal ceroid lipofuscinosis type 7. Segregation was confirmed in both parents, who were heterozygous. The neuronal ceroid lipofuscinoses are a group of diseases caused by mutations in 13 genes (*CLN1‐8*, *CLN10‐14*) and display an overlapping disease spectrum. For instance, recent proteomics analysis has revealed that one of the proteins which is markedly downregulated in neuronal ceroid lipofuscinosis type 7, is, besides MFSD8 itself, *CLN5* (Danyukova et al., 2018). Moreover, while for instance the *CLN1*,*CLN2, *and *CLN10* genes encode for proteins with an enzymatic activity, this has currently not been demonstrated for *CLN3* and *MFSD8*, which give rise to endosomal/lysosomal transmembrane proteins, consequently making the development of biochemical assays for these latter two challenging (Mohammed, O'Hare, Warley, Tear, & Tuxworth, 2017). Here, gene panel screening proves to be a valuable alternative strategy.

## 4 DISCUSSION

Prior to the usage of NGS, our lab performed standard biochemical analysis based on 4MU‐labeled substrates for the detection of LSDs. In total, biochemical tests were implemented in the clinic for 21 different LSDs (Table 2). During an evaluation period of 30 months (1,069 samples), a diagnostic yield of 4.58% was attained for this approach. The NGS methodology used here, results in a diagnostic yield of 15%. This increase in yield goes hand in hand with the fact that 51 genes are now being investigated in comparison to the 21 enzymes which were tested previously. This implies that, although the absolute yield has more than tripled, a less strong improvement is seen in relative terms. For instance, when looking to the gene panel results of the LSDs which are in our biochemical testing list, a yield of 9/150% or 6% could be observed, which is slightly higher, but comparable to the 4.58% of the biochemical tests. This indeed indicates that the increase in yield of the gene panel is mainly due to the additional LSDs which were added to the panel. However, we also detected several patients with a carriership status of certain genes, implying that the real diagnostic success rate could be higher due to the fact that deletions at the gene level cannot be detected. In these cases, biochemical testing is appropriate. The diagnostic yield of gene panels varies strongly according to the type of the disease for which the panel is offered. For instance, while the yield for a congenital glycosylation disorders gene panel was found to be 14.8% (Jones et al., 2013), this can be as much as 32% for a hypertrophic cardiomyopathy panel (Alfares et al., 2015). This indicates that the disease nature plays a major role: the clinical presentation of hypertrophic cardiomyopathy is for instance expected to be more clear than when dealing with patients with a suspicion of lysosomal storage diseases who generally present with a more aspecific phenotype. In view of this, one could argument for the implementation of whole‐exome sequencing which has been shown to obtain yields between 25% and 50% (Xue, Ankala, Wilcox, & Hegde, 2015). However, while the cost of whole‐exome sequencing is rapidly decreasing, trio‐analysis is advisable, and the data analysis is still more extensive than that of dedicated gene panels. Furthermore, it is expected to detect more unsolicited findings and variants of uncertain significance.

Interestingly, we could not observe a difference in diagnostic yield between children and adolescents on the one hand and adult patients on the other hand. This could imply that, despite an often more severe presentation of the phenotype in younger patients, the more aspecific nature of the disease at these ages potentially introduces a negative bias in terms of success rate. Conversely, patients in whom a late‐onset phenotype might present with a more specific phenotype, resulting in a better patient selection prior to LSD panel testing.

Taken together, we here demonstrate that the NGS‐based approach for the detection of LSDs is a valuable alternative next to the well‐established biochemical assays. The fact that a broader spectrum of diseases can be monitored in one single test significantly shortens the analysis time in complex cases and in cases where a biochemical test cannot be offered. Moreover, the genetic information is readily available, allowing familial segregation analysis. However, in case of a positive finding, biochemical testing still should be performed. This is especially the case when only a single mutation is detected or only variants of uncertain significance are observed in a particular gene. The gene panel can in these cases guide laboratories toward performing a specific biochemical test leading to a correct diagnosis.

In view of the possibility of detecting VUS and/or secondary findings, reporting of the results should be done carefully and should always be coupled to the clinical phenotype of the patient. For instance, when a pathogenic mutation is found in a gene which is not related to the clinical symptoms of the patient, the (probable) non‐causality of this mutation should be made clear in the report and the decision to report should always be in accordance with the informed consent papers signed by the patient. Nevertheless, reporting of this noncausal mutation could be worthwhile in terms of further familial testing.

The larger repertoire of diseases that can be interrogated by use of NGS panel testing mostly benefits patients in whom symptoms are not fully specific for a certain LSD. However, it has to be taken into account that large genomic deletions cannot be detected and that this gap should be filled in for genes where common deletions have been readily reported. In our case, we implemented a deletion test for *CTNS*, *CLN3, GAA,* and *GALC*. Furthermore, when dealing with clinical symptoms which are classical for a particular LSD, running a targeted biochemical test might be more appropriate, since NGS analysis will most likely require more resources.

We here thus show that NGS gene panel testing is a valuable alternative in comparison to the already established biochemical testing. By implementing the panel, we were able to broaden our disease spectrum and as such increased the absolute diagnostic yield. Furthermore, this methodology allows us to detect carriership status, allowing for further family testing and counseling. Taken together, we suggest to implement panel testing in the standard flow of LSD diagnostics.

## CONFLICT OF INTEREST

AG received supporting grants from Shire and Sanofi for the validation of the panel.
